# The Primary Mode of Action of *Lippia graveolens* Essential Oil on *Salmonella enterica* subsp. *Enterica* Serovar Typhimurium

**DOI:** 10.3390/microorganisms11122943

**Published:** 2023-12-08

**Authors:** Annie Rubio Ortega, Elodie Guinoiseau, Jean-Pierre Poli, Yann Quilichini, Dominique de Rocca Serra, Maria del Carmen Travieso Novelles, Ivette Espinosa Castaño, Oriela Pino Pérez, Liliane Berti, Vannina Lorenzi

**Affiliations:** 1 Laboratory of Chemical Ecology, Agricultural Pest Group, National Center for Animal and Plant Health, San José de las Lajas 32700, Mayabeque, Cuba; annie@censa.edu.cu (A.R.O.); mcarmen@censa.edu.cu (M.d.C.T.N.); oriela@censa.edu.cu (O.P.P.); 2 Projet Ressources Naturelles, UMR CNRS 6134 SPE, Université de Corse, BP 52, 20250 Corte, France; poli_jp@univ-corse.fr (J.-P.P.); quilichini_y@univ-corse.fr (Y.Q.); deroccaserra_d@univ-corse.fr (D.d.R.S.); berti_l@univ-corse.fr (L.B.); lorenzi_v@univ-corse.fr (V.L.); 3 Laboratory of Bacteriology, Microbiology Group, National Center for Animal and Plant Health, San José de las Lajas 32700, Mayabeque, Cuba; espinosa@censa.edu.cu

**Keywords:** botanical product, foodborne bacteria, cytoplasmic membrane, thymol, carvacrol

## Abstract

Essential oils are known to exhibit diverse antimicrobial properties, showing their value as a natural resource. Our work aimed to investigate the primary mode of action of Cuban *Lippia graveolens* (Kunth) essential oil (EO) against *Salmonella enterica* subsp. *enterica* serovar Typhimurium (*S. enterica* ser. Typhimurium). We assessed cell integrity through various assays, including time-kill bacteriolysis, loss of cell material with absorption at 260 and 280 nm, total protein leakage, and transmission electron microscopy (TEM). The impact of *L. graveolens* EO on membrane depolarization was monitored and levels of intracellular and extracellular ATP were measured by fluorescence intensity. The minimum inhibitory and bactericidal concentrations (MIC and MBC) of *L. graveolens* EO were 0.4 and 0.8 mg/mL, respectively. This EO exhibited notable bactericidal effects on treated cells within 15 min without lysis or leakage of cellular material. TEM showed distinct alterations in cellular ultrastructure, including membrane shrinkage and cytoplasmic content redistribution. We also observed disruption of the membrane potential along with reduced intracellular and extracellular ATP concentrations. These findings show that *L. graveolens* EO induces the death of *S. enterica* ser. Typhimurium, important information that can be used to combat this foodborne disease-causing agent.

## 1. Introduction

Foodborne diseases have important repercussions on public health, food safety, productivity, and poverty. Every year, almost 600 million people are infected by foodborne pathogens, killing 420,000 of them. Low and middle-income countries are the most affected, with estimated annual costs of 110 billion $ in productivity and commercial losses including treatment costs due to unhealthy food consumption [1]. With the phenomenon of climate change, there has been a significant increase in the risk to public health through its effects on microorganisms. The influence of this phenomenon on antimicrobial resistance and zoonotic diseases directly relates to food safety [2].

The genus *Salmonella* is one of the main pathogens causing foodborne diseases worldwide [3]. *Salmonella*, an anaerobic facultative Gram-negative bacillus, is a zoonotic pathogen that belongs to the Enterobacteriaceae family. *Salmonella enterica*, with more than 2500 serovars [4], shows a wide range of hosts including animals, humans, and plants. It can be found in the intestines of many animals of economic and food importance, such as pigs and poultry [5]. In plants, *S*. *enterica* ser. Typhimurium is not limited to the surface; it invades plant tissues and grows inside them, making washing or sterilizing processes useless in preventing human or animal infections [6,7]. These species can develop antibiotic resistance and form biofilms [8,9,10], putting them on the WHO’s «priority pathogen list» for the research and development of new antibiotics [11].

Essential oils (EO) are aromatic and volatile liquids with a complex composition. They can be obtained from fresh and dry plant material (i.e., flowers, roots, leaves, seeds, husks, fruits, or the whole plant) by different methods of extraction such as hydrodistillation, steam distillation, and cold press techniques [12,13]. Among the natural products of plant origin, EOs stand out for their versatility in biological properties. One of their main advantages is their antibacterial activity as broad-spectrum substances against Gram-positive and Gram-negative bacteria, including antibiotic-resistant strains [14,15]. 

Plants of the family *Verbenaceae* are known for their capacity to produce EOs with diverse uses. The genus *Lippia* includes more than 100 plant species that have been used in traditional Latin American medicine [16]. One of the major commercial species is *Lippia graveolens* (Kunth), an aromatic plant native to southern North America and largely distributed in warm temperate and tropical regions. Commonly known as “Mexican oregano”, it is consumed as a food seasoning in Central and South America. In addition to *L*. *graveolens* use in traditional medicine, EOs extracted from these plants are recognized for their numerous biological properties, such as their analgesic, antipyretic, anti-inflammatory, and antibacterial activities. The EO of this plant exhibits antibacterial activity against Gram-positive bacteria such as *Staphylococcus aureus* (ATCC 6538) and Gram-negative bacteria like *Escherichia coli* (ATCC 11229) or *Pseudomonas aeruginosa* (ATCC 9027), which have been related to major components like thymol, carvacrol, and *p*-cymene, which are already known to interfere with the plasmic membrane [17,18,19]. 

Studies on the antimicrobial activity of EOs are abundant throughout the world; however, more in-depth and integrative vision is needed to understand their effect on microbial cells. The goal of this work was to investigate the primary mode of action of the EO extracted from *L*. *graveolens* Cuban plants on *Salmonella enterica* subsp. *enterica* serovar Typhimurium.

## 2. Materials and Methods

### 2.1. Essential Oil 

The EO of *L*. *graveolens* was supplied by the Chemical Ecology Laboratory of the National Center for Animal and Plant Health, Cuba. The plant material was collected in January 2019 in the town of Jaruco, located in Jaruco, Mayabeque, Cuba (23°04′35.7″ N 81°57′51.4″ W) and was identified as *Lippia graveolens* (Kunth) by specialists from the “Liliana Dimitrova Horticultural Research Institute”. This essence was obtained by hydrodistillation of fresh material for 3 h using Clevenger equipment [20]. The EO of *L*. *graveolens* was dried with sodium sulfate (Merck, Madrid, Spain) and a yield of 1.94 ± 0.69 mL was obtained. This essence was previously characterized and its chemical composition was analyzed by GC-MS.

### 2.2. GC-MS Analysis

The analysis was carried out using an Agilent 6890 gas chromatograph (Agilent (Hewlett-Packard), Palo Alto, CA, USA) coupled with a 5973-quadrupole mass spectrometer (Agilent (Hewlett-Packard), Palo Alto, CA, USA) detection system (GC-MS) operated with a split/splitless injector. Fused silica capillary column: SPB-5; 15 m × 0.25 mm ID × 0.10 µm film thickness (Supelco, Bellefonte, PA, USA). Temperature program: from 60 °C (2 min) to 100 °C at 4 °C/min and from 100 °C to 250 °C (5 min) at 10 °C/min. Injection volume: 0.5 μL. Carrier gas: He, constant flow 1.0 mL/min. Injection mode split splitless: split (20:1). MS interface temp.: 280 °C; EI mode operating at 70 eV; mass range: 40–800 amu. Data handling was performed through MS ChemStation 1999. Compound identification was based on mass spectral comparison with those of the NBS-NISTASCI and Wiley275 databases.

### 2.3. Bacterial Strains and Growth Conditions

The *Salmonella enterica* subsp. *enterica* serovar Typhimurium strain ATCC 14028 (CIP 104115) was purchased from the Collection of the Institute Pasteur (CIP, Paris, France). Before each experiment, the strain was preserved frozen in cryovials at −80 °C and routinely grown at 37 °C on Mueller–Hinton 2 agar (MHA, Oxoid, Basingstoke Hampshire, UK).

### 2.4. Antimicrobial Susceptibility Testing

#### 2.4.1. Disc Diffusion Assays

The agar diffusion method was used to determine the antibacterial activities (Clinical and Laboratory Standards Institute—CLSI, 2018). The inoculum was prepared by diluting overnight cultures in Mueller–Hinton broth (MHB, Oxoid, Basingstoke Hampshire, UK) to 10^6^ CFU/mL. Filter paper discs (6 mm diameter, Dutscher, Bernolsheim, France) were placed onto the inoculated Petri dishes containing Mueller–Hinton 2 agar (MHA, Oxoid, Basingstoke Hampshire, UK) and 15 µL of the tested products were applied on them. After 1 h at room temperature, the plates were incubated at 37 °C for 24 h. The diameter of the inhibition zones was measured (mm) and recorded as mean ± standard deviation (SD). Each test was performed in triplicate in at least three separate experiments. Ciprofloxacin discs (5 µg, Bio-Rad, Hercules, CA, USA) were used as positive controls.

#### 2.4.2. Minimum Inhibitory Concentration and Minimum Bactericidal Concentration Assays

The minimum inhibitory concentration (MIC) is defined as the lowest sample concentration that leads to at least 90% inhibition of the initial bacterial inoculum. MIC assays were performed by a rapid INT (*p*-iodonitroterazolium chloride, Sigma-Aldrich) colorimetric assay [14]. The *L*. *graveolens* oil was serially twofold diluted in dimethylsulfoxide (DMSO, Sigma-Aldrich, Saint-Louis, MO, USA). The DMSO was previously tested for antibacterial activity, and no detrimental effect on bacterial growth was observed at the concentration used. The solutions obtained were then added (10 µL) to a 96-well microplate containing 190 µL of MHB (1:20, *v*/*v*) inoculated with 10^6^ CFU/mL. Microplates were incubated at 37 °C for 24 h. The MIC of the samples was then detected after adding (50 µL) of INT (0.2 µg/mL). The viable bacteria reduced the yellow dye to pink. All determinations were performed in triplicate and a negative control consisting of MHB with DMSO (5%, *v*/*v*) was systematically included. An inoculation loop was introduced in each well and grown on a Mueller–Hinton agar plate free of the antimicrobial agent to determine the minimum bactericidal concentration (MBC), which was defined as the lowest concentration of the oil resulting in a negative subculture.

### 2.5. Time-Kill Studies

Time-kill assay was performed according to the method described by Klepser et al. [21] and modified by Viljoen et al. [22]. The antibacterial activity of *L*. *graveolens* EO at its MIC on *S*. *enterica* ser. Typhimurium was determined by measuring the reduction of the number of CFUs (colony forming units) per milliliter at 0, 15, 30, 45, 60, 120, 180, 240 min, and 24 h of incubation at 37 °C with agitation. The tested product was applied at MHB with DMSO (0.1%) inoculated with 10^6^ CFU/mL. The inoculated medium containing DMSO and without EO was used as a control. At each evaluation time, 100 µL was collected and serially diluted in MHB; in each serial dilution step, 100 µL was transferred to two MHA plates in numbered sections and incubated at 37 °C for 24 h. CFUs were counted after incubation. This assay was performed in triplicate.

### 2.6. Cell Integrity Studies

A bacteriolysis assay was carried out according to the standard method described by Carson et al. [23]. A bacterial suspension, prepared by inoculating two colonies of *S*. *enterica* ser. Typhimurium from overnight cultures on MHA with 40 mL of MHB, was incubated at 37 °C for 24 h with shaking. After incubation, the bacteria were separated from the growth medium by centrifugation at 10,000× *g* (Hettich centrifuge, MIKRO 200R, Vlotho, Germany) for 12 min at 4 °C, washed twice with phosphate-buffered saline (PBS, pH 7.4), and resuspended in PBS supplemented with 0.01% Tween 80 (PBS-T, *v*/*v*). The bacterial suspension was adjusted so that the optical density (OD) at 550 nm of a 1:100 dilution was 0.310 (~3 × 10^8^ CFU/mL). *L*. *graveolens* EO was added to the bacterial suspension at the MIC. PBS-T was added to the control suspension. The suspensions obtained were mixed for 20 s with a vortex mixer. Samples (1 mL) were taken in duplicate every 30 min from 0 h to 2 h. They were centrifuged and the pellet was resuspended in 1 mL of PBS-T. The optical density at 620 nm (OD_620_) was measured immediately (Jasco UVisco UV-1200, Pfungstadt, Germany). This assay was performed in three independent experiments. The results were expressed as the ratio (in percent) of the OD_620_ at each sampling time over the OD_620_ at 0 min.

### 2.7. Loss of Cytoplasmic Material

The release of 260 nm and 280 nm of absorbing materials from *S*. *enterica* ser. Typhimurium cells treated with *L*. *graveolens* EO at MIC was determined by the bacterial suspension (10^8^ CFU/mL) in PBS supplemented with 0.01% Tween 80 (PBS-T, *v*/*v*) [14]. A suspension without EO was used as a control. The samples were incubated at 37 °C with shaking. They were taken at 0, 30, 60, 90, and 120 min, and centrifuged at 10,000× *g* for 12 min. The absorbance of the obtained supernatant was measured at 260 and 280 nm using a spectrophotometer (Jasco UVisco UV-1200, Germany). Each test was performed in three independent experiments. The results were expressed as the difference between the OD_260_ or OD_280_ at each sampling time and the OD_260_ or OD_280_ at time 0.

### 2.8. Determination of Released Proteins 

The total protein losses released by the action of *L*. *graveolens* EO on *S*. *enterica* ser. Typhimurium were determined by Lowry’s method [24]. The inoculum and samples were prepared as they were for the loss of cytoplasmic material. In total, 1% sodium lauryl sulfate (SDS) and bovine serum albumin (BSA) was used as a standard. After the treatment with the MIC, the samples were taken at 0 and 120 min and centrifuged at 10,000× *g* for 12 min. A suspension without EO was used as a negative control. Lowry’s method was used and the optical density was measured at 730 nm using a spectrophotometer (Jasco UVisco UV-1200 spectrophotometer, Germany). Each sample was prepared in triplicate, and three independent experiments were performed. The results were expressed as the mean ± standard error. 

### 2.9. Measurement of Intra- and Extracellular Adenosine 5′-Triphosphate (ATP) Concentrations

To determine the action of *L*. *graveolens* EO on energetic molecules, the intracellular and extracellular ATP concentrations were measured as described by Gill and Holley [25], with modifications by Turgis et al. [26]. The overnight cultures of *S*. *enterica* ser. Typhimurium were centrifuged at 10,000× *g* for 10 min and the supernatants were removed. The cell pellets were washed twice with 20 mM of phosphate potassium buffer (PPB, pH 7.0) and the cells were collected by centrifugation under the same conditions. A cell suspension (10^8^ CFU/mL) was prepared in PPB (20 mM; pH 7.4) with glucose (50 mM) and DMSO (0.1%). The EO at MIC. 30 mM of polymyxin B (PMB) was used as a positive control; the suspension without EO was used as a negative control. The suspensions were incubated at 37 °C for 7 min with agitation. Then, the samples were centrifuged at 10,000× *g* for 12 min and the supernatants were separated from the pellets. 

For the extracellular ATP, the supernatants were quickly placed on ice, and 50 µL of the samples was added to 96-well black plates in duplicate. Then, 50 µL of MIXED kit for ATP (ATP-kit, Sigma, Saint-Louis, MO, USA) were applied to each well, and the plate was incubated for 30 min on ice in the dark. Fluorescence was measured using a spectrofluorophotometer (Jasco, FP-83000 Jasco, Germany) with an excitation wavelength (λ_ex_) = 535 nm and excitation wavelength (λ_em_) = 587 nm. Each test was performed in three independent experiments. The results were expressed as the ratio (%) of the relative fluorescence unit (RFU) at each sampling time over the RFU of the DMSO control.

For the intra-cellular ATP, the pellets were resuspended in 1 mL of NaCl (0.85%) and centrifuged at 10,000× *g* for 10 min. The cells were resuspended in 200 µL of ATP buffer assays (ATP-kit, Sigma, Saint-Louis, MO, USA). Afterward, 5 µL of 15% cetyltrimethylammonium bromide (CTAB, Sigma) was added to each treatment and incubated at room temperature for 15 min. The samples were centrifuged and 50 µL of the supernatant was taken and applied to 96-well black plates in duplicate. Then, this experiment was continued as for the extracellular ATP assay. 

### 2.10. Membrane Depolarization Assay 

The cytoplasmic membrane depolarization activity of *L*. *graveolens* EO on *S*. *enterica* ser. Typhimurium was measured by using 3,3′-dipropylthiadicarbocyanine iodide (DiSC_3_(5), Sigma, Saint-Louis, MO, USA) [27]. An *S*. *enterica* ser. Typhimurium overnight culture in MHB was centrifuged at 10,000× *g* for 12 min at 4 °C, washed twice with phosphate-buffered saline (PBS, pH 7.4), and resuspended in a buffer solution (10 mM HEPES (4-(2-hydroxyethyl)-1-piperazineethanesulfonic acid) with 50 mM glucose (pH 7.0) and 0.1% DMSO). The bacterial suspension was adjusted at 10^8^ CFU/mL and 99 µL was applied to 96-well black plate in duplicate. The cells were incubated with 1.68 µM DiSC_3_(5) at room temperature with shaking for 1 h. Then, KCl was added to a final concentration of 0.1 M to equilibrate the cytoplasmic and external K^+^ and incubated for 5 min. An aliquot of 1 µL of the *L*. *graveolens* EO at MIC was applied, and 30 mM of PMB and 4 mM of CTAB were used as positive controls. A suspension without EO was used as a negative control. Fluorescence was monitored using a spectrofluorophotometer (Jasco, FP-83000, Germany) with an excitation wavelength (λ_ex_) = 622 nm and excitation wavelength (λ_em_) = 670 nm). The results were expressed in RFU. This assay was performed in three independent experiments.

### 2.11. Transmission Electron Microscopy (TEM) 

A suspension of *S*. *enterica* ser. Typhimurium in its exponential phase of growth was prepared by inoculating 80 mL of MHB and incubating it at 37 °C for 24 h with shaking [14]. The bacterial suspension was adjusted so that the OD_620_ of a 1:100 dilution in MHB was 0.2 (10^8^ CFU/mL) with Tween 80 (0.1%, *v*/*v*). The cells of *S*. *enterica* ser. Typhimurium were treated with the MIC of *L*. *graveolens* EO for 7 min. The negative control was the suspension without EO. After centrifugation at 10,000× *g* for 10 min, the pellets were first fixed by 2.5% glutaraldehyde in 0.1 M cacodylate buffer (pH 7.2) for 1 h at room temperature and then post-fixed in 2% osmium tetroxide in 0.1 M cacodylate buffer (pH 7.2) for 1 h at room temperature. The postfixed microbial pellets were processed in graded ethyl alcohol, propylene oxide, and Spurr resin and cured for 24 h at 45 °C. Ultrathin sections were stained with uranyl acetate followed by lead citrate and then examined with a transmission electron microscope (HITACHI H-7650, Tokyo, Japan) at an accelerating voltage of 80 kV.

### 2.12. Statistical Analysis 

All assays were performed in triplicate in the independent experiments and the data obtained were presented as the mean values and standard errors. Differences between the mean values of the inhibition zones, log (CFU/mL+1) viable cells, percentages of material release, and released proteins were tested for significance by using Student’s test for independent samples. The data obtained from cell integrity studies and measurements of ATP concentrations were statistically processed by a simple variance analysis, and the means were compared using Tukey multiple range tests with a significance level of 5%. The statistical package InfoStat/L 2018 was used and GraphPad Prism 9.0 was used to graph the data.

## 3. Results

### 3.1. Composition of Lippia graveolens EO of Plantas Cultivated in Cuba 

GC-MS analysis of the *Lippia graveolens* EO used in this study led to the identification of twelve components that account for 91.4% of the whole oil (Table 1). The composition of *L*. *graveolens* EO is largely dominated by oxygenated monoterpenes (71.43%). The hydrocarbonated fraction, made up of monoterpenes (γ-terpinene (7.35%), *p*-cymene (6.51%), α-terpinene (1.31%), and myrcene (1.30%)) and the sesquiterpene (E)-caryophyllene (3.50%), was present in appreciable amounts (19.97%). Among oxygenated components, the monoterpenols thymol (42.71%) and carvacrol (22.2%) were predominant. 

### 3.2. Susceptibility of Salmonella ser. Typhimurium to Lippia graveolens Essential Oil

The EO of *L*. *graveolens* inhibited the growth of the *S*. *enterica* ser. Typhimurium strain. A large zone of inhibition (56.67 mm ± 3.3) was revealed by the action of this EO. Indeed, according to Mazzarrino et al. [28], an inhibition diameter higher than 20.1 mm is related to strong antibacterial activity. The MIC and MBC values were, respectively, 0.4 mg/mL and 0.8 mg/mL, leading to an MBC/MIC ratio of 2. As this is lower than 4, the activity of *L*. *graveolens* EO on the *S*. *enterica* ser. Typhimurium strain is considered to be bactericidal.

### 3.3. Time-Kill Studies

**Figure 1:**
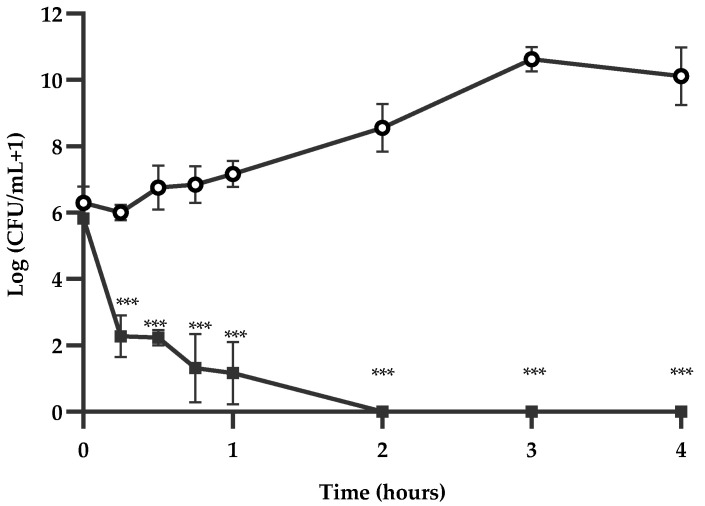
Time-kill curves of *S. enterica* ser. Typhimurium treated with *Lippia graveolens* essential oil (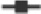) at MIC and untreated cultures (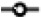). Mean values of triplicate independent experiments and standard deviations are shown. *** Significant differences between treatments compared to the control (*p* < 0.0001).

*Lippia graveolens* EO showed a negative kinetic effect on *S*. *enterica* ser. Typhimurium growth, with reduced viability with the MIC dose (Figure 1). This EO reached the bactericidal end-point (99.9% or ≥3 log10 of inhibition [29]) in only 15 min. Total inhibition was accomplished after 2 h of treatment and was sustained for 24 h.

### 3.4. Cell Integrity Studies

**Figure 2:**
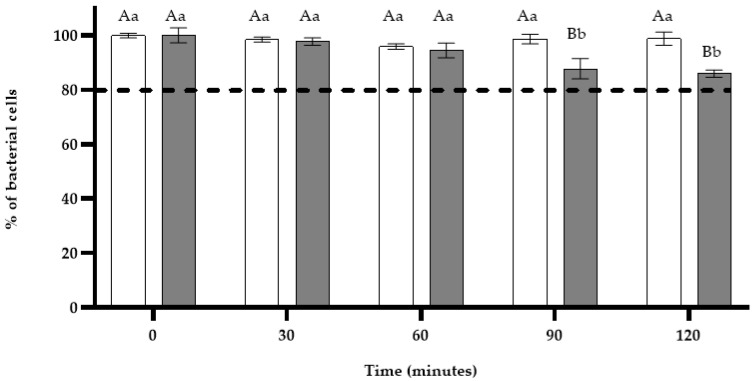
Cell integrity of *S. enterica* ser. Typhimurium-untreated strain (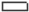) and after treatment with *L. graveolens* essential oil (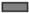) at MIC. Mean values of triplicate independent experiments and standard deviations are shown. Different capital letters indicate significant differences (*p* < 0.05) in treatments at specific times and different lowercase letters show significant differences (*p* < 0.05) in the time of a specific treatment.

The OD_620_ of negative controls did not significantly vary from the initial absorbance after 120 min. As shown in Figure 2, the treatment of *S*. *enterica* ser. Typhimurium cells by *L*. *graveolens* EO at its MIC did not alter cell viability in the first 30 min. From 60 min to 120 min, a non-significant decrease in the OD_620_ is observed (OD_620_ nm ranged over means of 85 to 91% of cell viability). As cell integrity was higher than 80%, no bacteriolysis occurred.

### 3.5. Loss of Intracellular Material

*Lippia graveolens* EO at its MIC dose induced the release of 260 nm and 280 nm absorbing materials from *S*. *enterica* ser. Typhimurium (Table 2). Nucleic acids and proteins are among the cytoplasmic content released under the EO action. The loss of absorbent material, at both 260 and 280 nm, was observed to be significant (*p* < 0.05) after 30 min of exposure to the oil. The maximum proportion was obtained after 2 h of treatment; however, it was less than 15%. To correlate the release of cellular components by *L*. *graveloens* EO treatment, total protein contents were determined. This test was carried out at T0 and at the maximum release of cellular content time (2 h); no significant changes occurred between the oil treatment (0.35 ± 0.27 mg/mL) and the control (0.04 ± 0.02 mg/mL) (*p* < 0.05) at the time of exposure. 

### 3.6. Membrane Depolarization Assay

**Figure 3:**
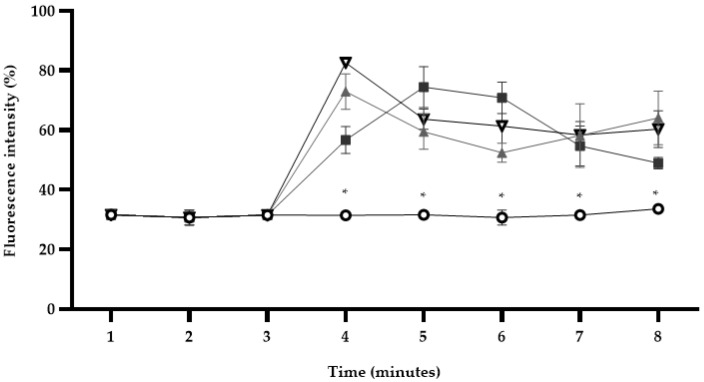
Depolarization of the cytoplasmic membrane of cells of *S. enterica* ser. Typhimurium treated with *Lippia graveolens* essential oil at MIC (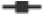), with 30 mM PMB (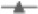) and 4 mM CTAB (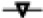) monitored by fluorescence intensity change. Untreated cells were used as negative controls (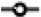). Mean values of triplicate independent experiments and standard deviations are shown. * Significant differences between treatments compared to the control (*p* < 0.05).

After the time required for fluorescence stabilization (3 min), *S*. *enterica* ser. Typhimurium cells were treated with *L*. *graveolens* EO used at its MIC. Following the addition of this treatment, the fluorescence of the DiSC_3_(5) probe used in this assay immediately increased from a mean value of 31.5 to 56.7 relative fluorescence units (RFUs) in 5 min (Figure 3). Similar results were obtained for both positive controls, PMB (from 31.5 to 59.7 RFUs) and CTAB (from 31.5 to 60 RFUs), while fluorescence from untreated cells stayed similar (from 30.7 to 33.6 RFUs).

The treatment was added after two min of equilibration.

### 3.7. Measurement of Intra- and Extracellular Adenosine 5′-Triphosphate (ATP) Concentrations

**Figure 4:**
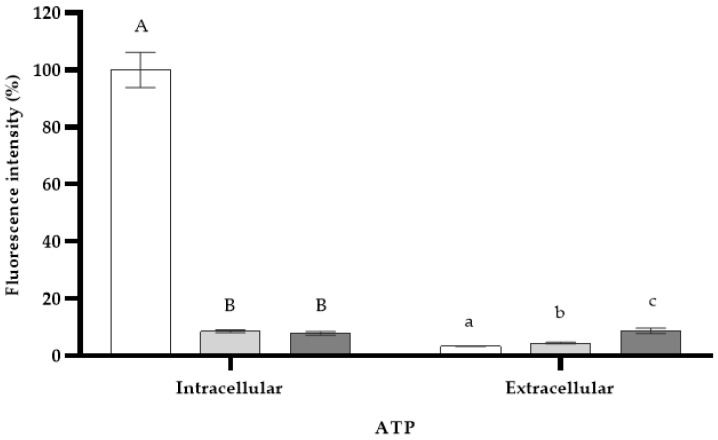
Intracellular and extracellular ATP concentrations of *S. enterica* ser. Typhimurium cells after 7 min of *Lippia graveolens* essential oil treatment at MIC (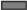). DMSO (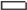) and PMB (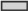) were used as controls. Mean values of triplicate independent experiments and standard deviations are shown. Different capital letters indicate significant differences (DF = 2; F = 221.1; *p* < 0.0001) in intracellular ATP and different lowercase letters show significant differences (DF = 2; F = 23.0; *p* = 0.0015) in extracellular ATP.

Measurements of intracellular and extracellular ATP were determined in *S*. *enterica* ser. Typhimurium cells after a seven-minute treatment with *L*. *graveolens* EO at its MIC (Figure 4). The EO (7.8% of RFUs) reduced intracellular ATP significantly (*p* < 0.05), similarly to the antibiotic polymyxin B (8.6% of RFUs). The concentration of extracellular ATP was very low (less than 10% of RFUs) regardless of the treatment used, and significant differences were evident between treatments (*p* < 0.05).

### 3.8. Transmission Electron Microscopy (TEM)

**Figure 5:**
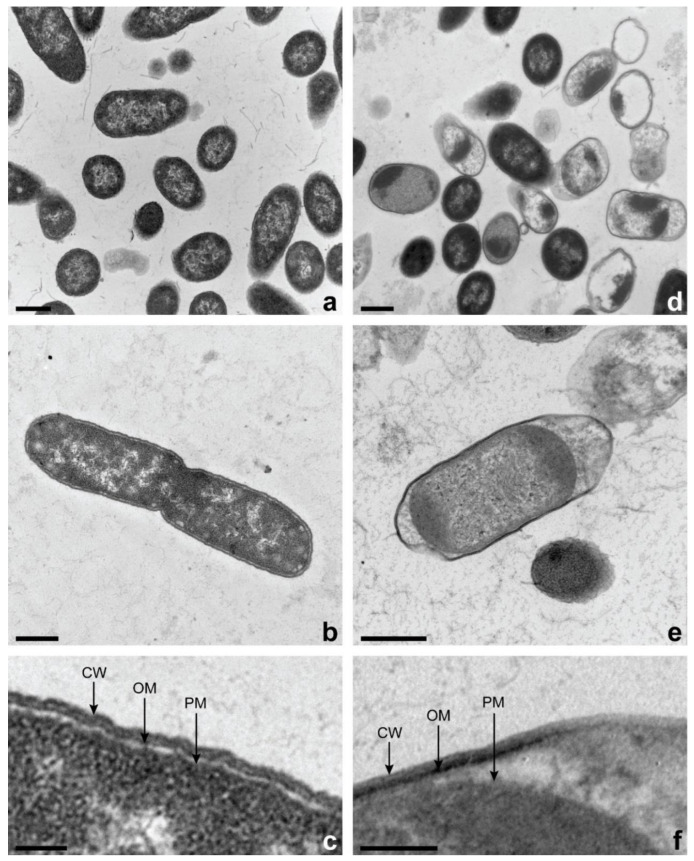
Transmission electron micrographs of stained *S. enterica* ser. Typhimurium cells. (**a**,**b**) Control cells with 500 nm scale bar and (**c**) 100 nm scale bar. (**d**,**e**) *S. enterica* ser. Typhimurium cells treated with *L. graveolens* oil at the MIC with 500 nm scale bar and (**f**) 100 nm scale bar. CW: cell wall; OM: outer membrane; PM: plasmic membrane.

*Salmonella enterica* ser. Typhimurium cells untreated (control: Figure 5a–c) and treated with *L*. *graveolens* EO at its MIC (Figure 5d–f) were observed by TEM. The cell wall and the plasmic membrane of *S*. *enterica* ser. Typhimurium control cells were regular and their cytoplasmic content was homogenous (Figure 5a–c). Strikingly, *L*. *graveolens* EO affected the cell integrity of *Salmonella* cells after only 7 min of exposure. Most of the cells conserved their morphology; however, their ultrastructure appeared to be different. Indeed, the cytoplasmic membrane of some cells was separated from the outer membrane. Moreover, the distribution of cytoplasmic content seemed to be less homogenous as aggregates were retrieved near the outer membrane (Figure 5d–f). 

## 4. Discussion

The main purpose of this work was to investigate the in vitro primary mode of action of *Lippia graveolens* (Kunth) EO on *Salmonella enterica* subsp. *enterica* serovar Typhimurium. The results from disc diffusion assay as well as the MIC (0.4 mg/mL) and MBC (0.8 mg/mL) determination values illustrate a potent and consistent inhibitory action of *L*. *graveolens* EO against this foodborne pathogen. EOs may inhibit the growth of bacterial cells (bacteriostatic) or destroy them (bactericidal). In our study, the displayed effect of *L*. *graveolens* EO on *Salmonella* cells appeared to be bactericidal. At the MIC dose, irreversible damage to cell viability was sustained in a short time (15 min). Similar results were obtained by Chauhan et al. [30]. They highlighted that treatment with thymol (0.750 mg/mL) reduced the number of *Salmonella enterica* serovar Typhimurium cells after 20 min of exposure. 

It is well known that the antibacterial activity of EOs is strictly related to their chemical composition. Their hydrophobic nature can interact with the lipidic membrane of bacterial pathogens, resulting in the leakage of the inner cell components and damage to the potassium ion reflux, finally leading to cell death [31]. Membrane disruptions affecting the structural stability of the membrane or changing its permeability are the main membrane modifications due to the action of EOs [32]. Generally, phenolic compounds are responsible for the major bactericidal effect of EOs on foodborne pathogenic bacteria. Their effectiveness is generally correlated to the occurrence of their major components. The phenolic compounds known as the most active ones disrupt the cell membrane as well as effectively inhibit the functional properties of the cell and, eventually, leak the inner materials of the cell [33]. 

The *Lippia graveolens* EO used in this study mainly consisted of thymol (42.7%), carvacrol (22.2%), γ-terpinene (7.3%), and *p*-cymene (6.5%). These moleculesweare also the major constituents of the *L*. *graveolens* EO cultivated in Mexico (carvacrol 43.7%, thymol 10.43%, *p*-cymene 6.4%) [12] and Rio de la Virgen-Jutiapa in Guatemala (carvacrol 44.8%, *p*-cymene 21.8%, thymol 7.4%) [34]. According to the chemical composition of the essential oil of *L*. *graveolens*, four chemotypes have been described: thymol (>50%), carvacrol (>50%), mixed thymol–carvacrol (>50% adding both components), and a fourth chemotype designated as sesquiterpene (with a predominance of α and β-caryo-phyllene). The essences with the greatest antibacterial activity correspond to the chemotypes that abound in monoterpene phenols [35].

Thymol and carvacrol are naturally occurring phenol monoterpene derivatives of *p*-cymene. They only differ by the position of their hydroxyl group on the benzene ring. Terpenes with a phenolic OH group are known to cause damage to cell function and structure due to their being highly lipophilic. They have a remarkable ability to be absorbed by the cell membrane, thus causing destabilization of the phospholipid bilayer. Once inside the intracellular medium, these compounds may bind to molecules like ATP or monovalent cations, such as K^+^, and actively transport them out of the bacterial cell. This process significantly disrupts the membrane potential and homeostasis of the cell [36,37]. Moreover, the hydroxyl group of these terpenes exhibits the capacity to bind to and inhibit proteins like ATPase [25].

Actual literature data show that the antibacterial mechanism reported for thymol and carvacrol isomers is involved in the disruption of bacterial membranes. It most frequently leads to bacterial lysis and leakage of intracellular contents inducing cell death [38]. Unlike its derivatives, *p*-cymene features a benzene ring without any hydroxyl group. Several studies state that non-oxygenated terpenes, specifically hydrocarbons such as limonene, terpinene, camphene, or α-pinene, exert weak antimicrobial action. When used on its own, *p*-cymene also demonstrates limited efficacy as an antimicrobial agent. Nevertheless, it enhances the activity of compounds such as carvacrol when used in combination, facilitating its penetration into bacterial cells. Moreover, its high affinity for lipidic bi-layers does not alter membrane permeability but seems to provoke a disruption in membrane potential [32,39]. The cell membrane mediates many processes such as energy conversion, nutrient processing, the synthesis of structural macromolecules, and the secretion of growth regulators [40]. For this, this cell structure is considered an important site of action for many EO constituents [32].

According to our results, the *L*. *graveolens* EO seems to cause the membrane destabilization of *S*. *enterica* ser. Typhimurium cells rather than their total destruction. Indeed, no cell lysis occurred until 2 h of treatment, which was consistent with the outflow of little absorbent cytoplasmic matrix at 260 nm and 280 nm. The DiSC_3_(5) fluorescent probe used to perform the membrane depolarization assay indicated that the *L*. *graveolens* EO interacts with the cytoplasmic bi-layer by disturbing the membrane potential of *S*. *enterica* ser. Typhimurium cells. Indeed, the EO exerted a comparable effect to the positive controls polymyxin B and CTAB, both known for disorganizing and permeabilizing cell membranes [41]. Crucial to initiate cell lysis, these two cationic surfactants cause significant damage when used in sufficient concentrations. In our study, protein released assays as well as intra- and extracellular ATP pool measurements rather suggested that the membrane permeability of *S*. *enterica* ser. Typhimurium cells is not altered by *L*. *graveolens*. ATP is used in vital cell processes that require energy, such as respiration, survival, growth, and replication. Other functions of ATP include signaling function and participation in storing and supplying energy in metabolism and enzymatic reactions. Compared to the negative control, no ATP was detected inside the cells when treated with the *L*. *graveolens* EO. However, this drastic decrease in the ATP pool inside the cell could not be correlated to the one found in the extracellular medium. 

The depletion of the internal ATP pool following the addition of lipophilic components, used alone or in combination, has already been reported. In *S*. *aureus* cells, the intracellular ATP concentration vanished by using the alcohol fraction extracts of *Cistus ladaniferus* L. EO without any increase in the external ATP pool [14]. As suggested by Ultee et al. [39], carvacrol seems to act as a proton exchanger able to deplete the proton motive force, thereby collapsing the ATP pool in *Bacillus cereus* cells in less than seven minutes. Cell respiration depends on the respiratory chain in the plasma membrane. This converts redox energy into an electrochemical gradient of protons (proton motive force), which subsequently drives ATP formation from ADP and phosphate by ATP synthase. The membrane potential and the transmembrane proton gradient are therefore strictly connected to the ATP synthesis pathway. The disturbance of one these parameters can thereby affect ATP synthase and damage the respiratory chain in cell respiration [42]. Thus, the loss of the internal ATP pool under *L*. *graveloens* EO treatment could be attributed to a reduction in ATP synthesis and/or an increase in its hydrolysis correlated with the disruption of the membrane potential detected. These phenomena are consistent with our TEM observations showing that the *L*. *graveolens* EO targeted the plasmic membrane of *S*. *enterica* ser. Typhimurium cells. Even if the cell remained morphologically intact, changes occurred inside the bacteria. Indeed, the cytomembrane separated from the outer membrane and differences in the electron-dense structure of cytoplasmic material were observed. These phenomena can be related to the capacity of EO to diffuse, penetrate, and disorganize the lipid tail region of the membrane [43]. 

Diverse research has already pointed out changes in the external morphology of *Salmonella* strains by the action of EOs. Raybaudi-Massilia et al. [44] described damages to the cell membrane of *Salmonella* Enteritidis by the action of lemongrass EO including its disruption and leakage of cell content. *Zataria multiflora* Boiss. EO triggered important morphological damages in *Salmonella* Typhimurium, such as an increase in the permeability and disruption of membranes [45]. As supported by our TEM micrographs, the *L*. *graveolens* EO action was also focused on the *S*. *enterica* ser. Typhimurium cytomembrane, which underwent shrinkage under the effect of the treatment.

Collectively, our findings suggest that the bactericidal activity of *L*. *graveolens* EO stems from its capacity to alter the cytoplasmatic membrane of *S*. *enterica* ser. Typhimurium, and the damage that occurs to this target seems to interfere with the energetic metabolic process of this bacterium. Due to the numerous alterations induced and the wide array of chemical constituents present in EOs, it is most likely that the effects induced by the *L*. *graveolens* EO cannot be attributed to a single specific mechanism. Therefore, an in-depth investigation is necessary to grasp the manner in which this oil and its active components interact with the bacterial membrane and the resulting implications at the cellular level. Understanding the mechanisms of action of antimicrobials is key to preventing resistance to these molecules. The complexity of EO chemical composition allows for different antimicrobial modes of action, not only at a particular location, like antibiotics do, but also at different cell sites. As a result, EOs are experiencing renewed interest as a substitute for antibiotics. Similarly, their proven efficacy on foodborne pathogens encourages the exploration of research paths that can support their utilization as natural food preservatives.

## 5. Conclusions

This study highlights the potential of using *Lippia graveolens* EO extracted from Cuban plants as a natural alternative for controlling *Salmonella enterica* ser. Typhimurium.

At a MIC dose of 0.4 mg/mL, the EO exhibited a bactericidal effect on this strain, leading to cell death within a few minutes. The active essence targeted the lipidic bi-layer of *Salmonella enterica* ser. Typhimurium by affecting its structure and also disturbing the membrane potential. These modifications seem ultimately to interfere with the energetic metabolic process of *Salmonella enterica* ser. Typhimurium. Further studies are required to fully comprehend whether other structures or specific metabolic pathways are affected by this EO.

## Figures and Tables

**Table 1:** Principal components of essential oil derived from *Lippia graveolens* plants cultivated in Cuba determined by GC-MS analysis.

Component	Retention Time (min)	Class of Compounds	Relative Abundance (%)	Match Quality
myrcene	2.82	mh	1.3	96
α-terpipene	3.22	mh	1.3	98
*p*-cymene	3.38	mh	6.5	93
1,8-cineole	3.49	om	4.0	99
γ terpinene	3.92	mh	7.3	96
terpinen-4-ol	5.80	om	1.1	97
α-terpineol	6.57	om	1.4	91
thymol	8.16	om	42.7	97
carvacrol	8.37	om	22.2	92
(E)-caryophyllene (=β-caryophyllene)	9.93	sh	3.5	99

mh: monoterpene hydrocarbon, om: oxygenated monoterpene, sh: sesquiterpene hydrocarbon.

**Table 2:** Percentage of material released at 260 nm and 280 nm into the extracellular medium of *S. enterica* ser. Typhimurium cells treated with *Lippia graveolens* essential oil at MIC.

Wavelength	Time (min)	Released Material (%)	
		Control	*Lippia graveolens* (0.4 mg/mL)
260 nm	0 30 60 90 120	0.00 ± 0.00 a 0.00 ± 0.00 a 0.01 ± 0.04 a 0.01 ± 0.04 a 0.00 ± 0.00 a	0.00 ± 0.00 a 3.40 ± 1.17 b 7.37 ± 2.80 b 9.24 ± 3.56 b 11.10 ± 4.32 b
280 nm	0 30 60 90 120	0.00 ± 0.00 a 0.11 ± 0.13 a 0.40 ± 0.20 a 0.30 ± 0.19 a 0.11 ± 0.13 a	0.00 ± 0.00 a 1.80 ± 0.49 ab 4.50 ± 1.62 b 7.50 ± 2.85 b 9.20 ± 3.55 b

Means ± standard deviations. Those in the same row with no letters in common are significantly different (*p* < 0.05).

## Data Availability

All the data generated or analyzed during this study are included in this published article.
